# H_2_S restores the cardioprotective effects of ischemic post-conditioning by upregulating HB-EGF/EGFR signaling

**DOI:** 10.18632/aging.101866

**Published:** 2019-03-26

**Authors:** Yuanzhou Zhang, Jun Gao, Weiming Sun, Xin Wen, Yuxin Xi, Yuehong Wang, Can Wei, Changqing Xu, Hongzhu Li

**Affiliations:** 1 Department of Pathophysiology, Harbin Medical University, Harbin, China; 2 Department of Osteology, the First Hospital of Harbin, Harbin, China; 3 The Key Laboratory of Cardiovascular Medicine Research, Harbin Medical University, Ministry of Education, Harbin, China; * Equal contribution

**Keywords:** hydrogen sulfide, post-conditioning, HB-EGF/EGFR pathway, aged cardiomyocytes, H9C2 cells

## Abstract

Hydrogen sulfide (H_2_S) reduces ischemia/reperfusion (I/R) injury and apoptosis and restores the cardioprotective effects of ischemic post-conditioning (PC) in aged cardiomyocytes by inhibiting oxidative stress and endoplasmic reticulum stress and increasing autophagy. However, the mechanism is unclear. In the present study, we observed a loss of PC-mediated cardioprotection of aged cardiomyocytes. NaHS (a H_2_S donor) exerted significant protective effects against H/R-induced cell damage, apoptosis, production of cleaved caspase-3 and caspase-9, and release of cytochrome c. NaHS also reversed the H/R-induced reduction in cell viability and increased HB-EGF expression, cellular HB-EGF content, and EGFR phosphorylation. Additionally, NaHS increased expression of Bcl-2, c-myc, c-fos and c-jun, and the phosphorylation of ERK1/2, PI3K, Akt and GSK-3β. PC alone did not provide protection to H/R-treated aged cardiomyocytes, but it was significantly restored by supplementation of NaHS. The beneficial effects of NaHS during PC were inhibited by EGFR knockdown, AG1478 (EGFR inhibitor), PD98059 (ERK1/2 inhibitor) or LY294002 (PI3K inhibitor). These results suggest that exogenous H_2_S restores PC-mediated cardioprotection by up-regulating HB-EGF/EGFR signaling, which activates the ERK1/2-c-myc (and fos and c-jun) and PI3K-Akt- GSK-3β pathways in the aged cardiomyocytes.

## Introduction

Myocardial ischemia is a leading cause of morbidity and mortality worldwide [1]. The first choice for treatment of acute myocardial ischemia is reperfusion therapy, which reduces infarct size by restoring oxygen to the myocardium [2]. However, it can also cause tissue damage due to the oxidative stress in can induce. This is known as ischemia/reperfusion (I/R) injury [3]. To reduce I/R injury, ischemic post-conditioning (PC) is used to reduce oxidative stress and mitochondrial permeability transition pore (mPTP) opening and to increase autophagy [4]. With aging, myocardial cell degeneration occurs as a result of increasing oxidative damage, decreasing mitochondrial function, declining gene/protein expression, changing DNA levels, loss of autophagic activity, and reductions in endogenous protective substances [5–8]. In addition, the beneficial effects of PC in ischemic myocardial cells may be weakened or even lost [9].

Heparin-binding epidermal growth factor (HB-EGF) is a member of the EGF family [10]. Acting via EGF receptor (EGFR), HB-EGF plays an important role in various tissues and organs [11], including the cardiovascular system [12]. For example, activation of HB-EGF/EGFR signaling decreases I/R injury and heart failure by promoting cardiac remodeling and inhibiting apoptosis, but increases atherosclerosis by stimulating the proliferation and migration of smooth muscle cells (SMCs) [13,14]. Kinases downstream of HB-EGF/EGFR catalyze the phosphorylation/activation of multiple cellular proteins and intracellular signaling molecules, including phosphatidyl inositol 3-kinase (PI3K), protein kinase B (Akt), and mitogen-activated protein kinase (MAPK) [15], which has a profound impact on cell growth, differentiation, migration, and apoptosis [15].

Hydrogen sulfide (H_2_S) is traditionally characterized as an environmental pollutant [16]. In recent years, however, research have shown that H_2_S is naturally produced in mammalian tissues and cells, and plays a role in many important physiological functions [16–19]. H_2_S can be endogenously generated by cystathionine gamma-lyase (CSE), cystathionine betasynthase (CBS), or 3-mercaptopyruvate sulfurtransferase (MST) with L-cysteine and homocysteine serving as the primary substrates [20]. Among these, CSE is the key enzyme mediating endogenous H_2_S production in the cardiovascular system [19], where it may inhibit the occurrence of such cardiovascular diseases as atherosclerosis, hypertension, heart failure and I/R injury [21].

We previously reported that exogenous H_2_S restores the cardioprotective effects of PC in isolated aged rat hearts and aged cardiomyocytes by mediating the PC-induced suppression oxidative stress, endoplasmic reticulum stress and mPTP opening while increasing autophagy [22–27]. However, the role played by the HB-EGF/EGFR signaling in the beneficial effects of H_2_S on PC-mediated cardioprotection is unclear. To examine that possibility, we induced aging in the H9C2 cardiomyocyte line using hydrogen peroxide (H_2_O_2_) and exposed the aged cells to hypoxia/reoxygenation (H/R) and PC protocols. We then investigated the effects of exogenous H_2_S on the recovery of PC-mediated cardioprotection and its possible mechanisms, including the role of HB-EGF/EGFR signaling and its downstream mediators (ERK1/2 and PI3K-Akt- GSK-3β) in the aged cardiomyocytes.

## RESULTS

### H_2_S increases HB-EGF levels and EGFR phosphorylation in aged H9C2 cells

**Figure 1:**
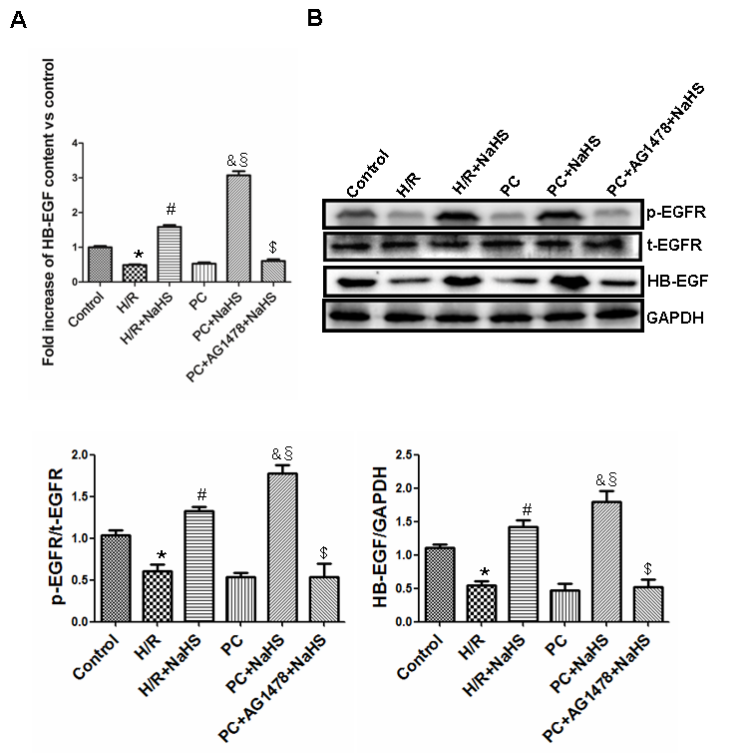
**Exogenous H_2_S increases the expression and content of HB-EGF and the level of phosphorylated EGFR in the aged H9C2 cells.** (**A**) The content of HB-EGF was measured using an HB-EGF ELISA kit. The HB-EGF content in the control group was considered the basal levels, and the HB-EGF content of the others was detected as the fold change from the control group. All data are the means ± S.E.M. of 8 determinations. (**B**) The activity of phosphorylated EGFR and the expression of HB-EGF. The graphs represent the optical density of the bands of phosphor-EGFR (p-EGFR) normalized to the expression of total-EGFR (t-EGFR). The graphs represent the optical density of the bands of HB-EGF normalized to the expression of the GAPDH signal. All data were from three independent experiments. * p<0.05 *vs*. control group; # p<0.05 *vs*. H/R group; & p<0.05 *vs*. PC group; § p<0.05 *vs*. H/R + NaHS group; $ p<0.05 *vs*. PC + NaHS group.

H/R markedly reduced HB-EGF expression and content in aged H9C2 cells, and that effect was reversed by treatment with NaHS. H/R also reduced EGFR levels, and that effect too was reversed by NaHS. The suppressive effects of H/R on HB-EGF and EGFR in the PC group were similar to those in the H/R group. Addition of NaHS to cells in the PC group significantly (p<0.05) increased HB-EGF and EGFR, and their levels the PC + NaHS group were higher (p<0.05) than in the H/R + NaHS group. The beneficial effects of PC + NaHS on HB-EGF and EGFR levels were inhibited by AG1478 (an EGFR antagonist), suggesting they were mediated via HB-EGF/EGFR signaling (Figure 1).

### H_2_S decreases aged H9C2 cell damage

**Figure 2:**
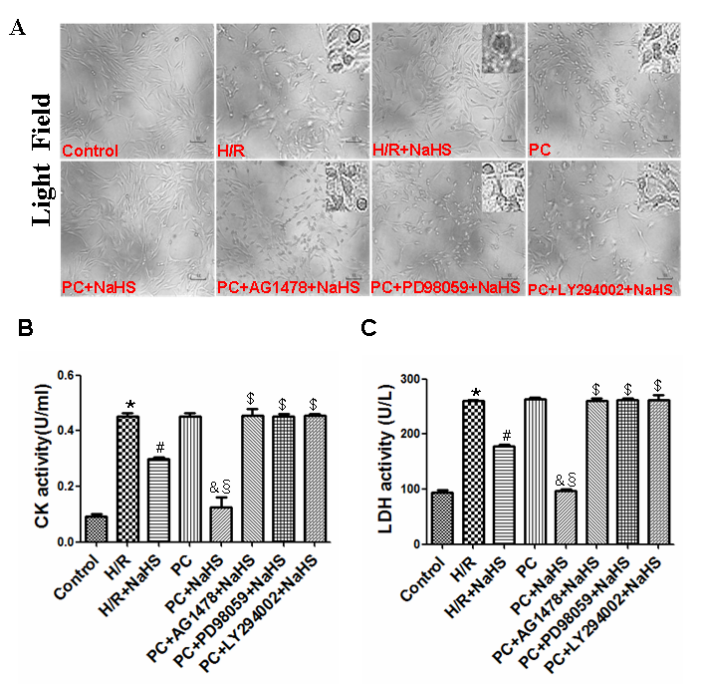
**Exogenous H_2_S decreases cell damage in the aged H9C2 cells.** (**A**) Exogenous H_2_S decreased cell damage. The cells were cultured in glass-bottom dishes and observed using a general inverted microscope (magnification ×100). (**B, C**) Exogenous H_2_S inhibited the activity of LDH (C) and CK (B). The LDH and CK activities were detected in the cell culture fluid. Data are the means ± S.E.M. of 8 determinations. * p<0.05 *vs*. control group; # p<0.05 *vs*. H/R group; & p<0.05 *vs*. PC group; § p<0.05 *vs*. H/R + NaHS group; $ p<0.05 *vs*. PC + NaHS group.

The photomicrographs in Figure 2A show that H9C2 cardiomyocytes in the control group were intact, granule-free, and grew in clusters. In the H/R group, the cell-to-cell adhesion among cardiomyocytes was lower, and the cells were more dispersed. In addition, the cells appeared collapsed. Compared with the H/R group, the cellular morphology of cardiomyocytes in the H/R + NaHS group was much improved. The appearance of cells exposed to H/R in the PC group was similar to that in the H/R group – i.e. PC alone had little protective effect against H/R in these aged cells. On the other hand, the beneficial effect of PC + NaHS on the morphology of cells exposed to H/S was even stronger than that of NaHS alone. This beneficial effect of PC + NaHS was inhibited by AG1478, PD98059 (an ERK1/2 inhibitor) or LY294002 (a PI3K inhibitor). Figure 2B and C show that H/R induced similar significant (p<0.05) increases in lactate dehydrogenase (LDH) and creatine kinase (CK) activities in untreated cells and cells treated with PC. Addition of NaHS to the PC-treated cells exposed to H/R led to marked (p<0.05) decreases in both LDH and CK activities. This inhibitory effect of PC + NaHS on LDH and CK activities was blocked by AG1478, PD98059, or LY294002.

### H_2_S enhances cell viability and inhibits apoptosis in aged H9C2 cells

**Figure 3:**
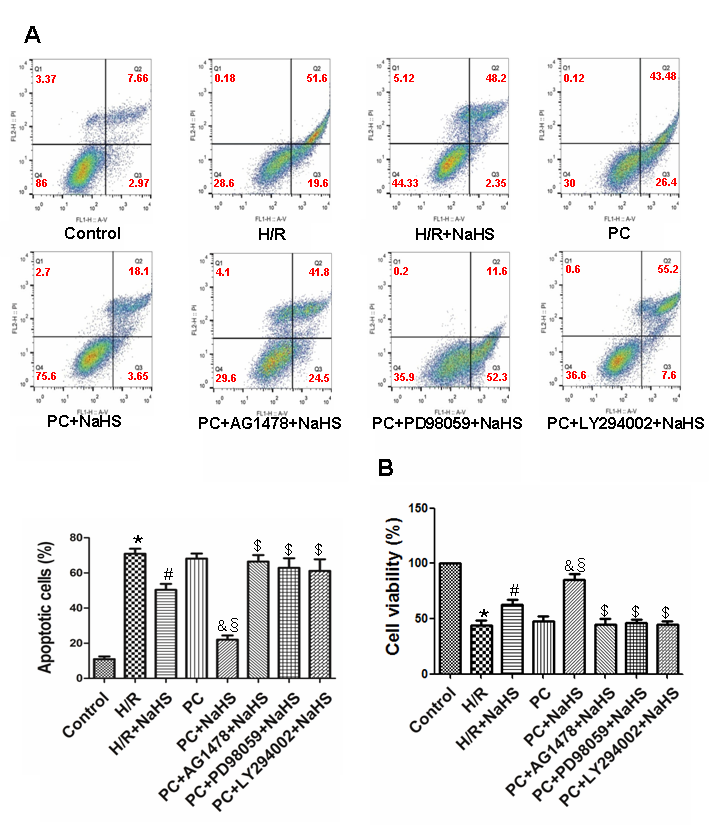
**Exogenous H_2_S decreases the apoptotic rate and increases cell viability in the aged H9C2 cells.** (**A**) Apoptosis was analysed by flow cytometry. The apoptotic rate = early apoptotic rate + late apoptotic rate. All data were from four independent experiments. (**B**) Cell viability measured using a CCK-8 kit. The viability of control cells was considered 100%, and the others were expressed as percentages of control. All data are the means ± S.E.M. of 8 determinations. * p<0.05 *vs*. control group; # p<0.05 *vs*. H/R group; & p<0.05 *vs*. PC group; § p<0.05 *vs*. H/R + NaHS group; $ p<0.05 *vs*. PC + NaHS group.

**Figure 4:**
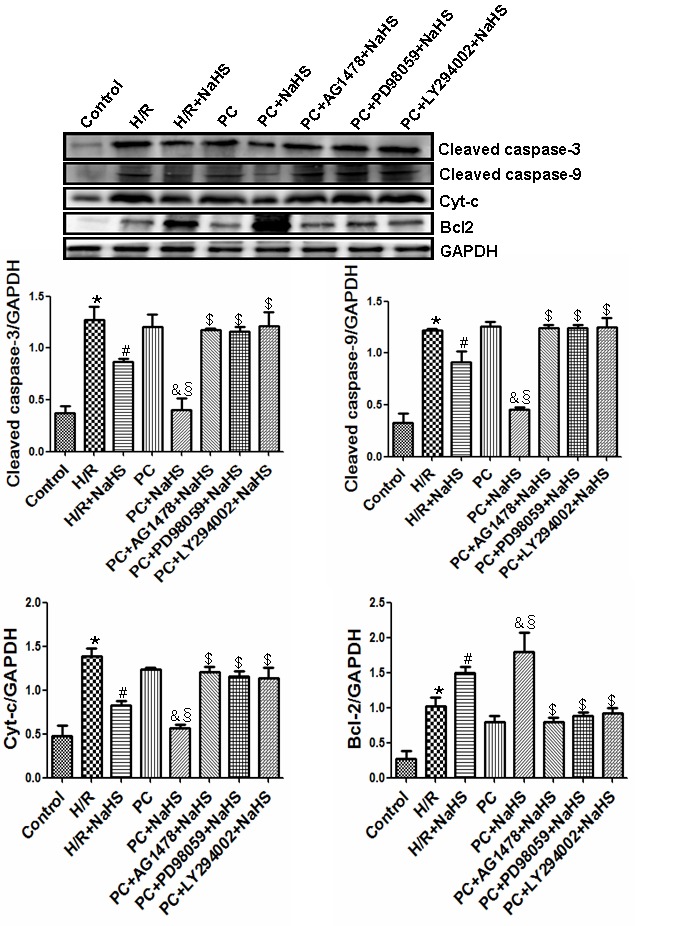
**Exogenous H_2_S inhibits the expression of cleaved caspase-3, cleaved caspase-9, and Cyt-c and promotes the expression of Bcl-2.** The intensity of each band was quantified by densitometry, and the data were normalized to the GAPDH signal. All data were from three independent experiments. * p<0.05 *vs*. control group; # p<0.05 *vs*. H/R group; & p<0.05 *vs*. PC group; § p<0.05 *vs*. H/R + NaHS group; $ p<0.05 *vs*. PC + NaHS group.

Compared to control cells, the viability of H9C2 cells exposed to H/R was reduced and the apoptotic rate was increased, as were levels of cleaved caspase-3, cleaved caspase-9, Cyt-c, and Bcl-2 (p<0.05) (Figures 3 and 4). H/R had similar effects on cells in PC group. However, those adverse effects of H/R were significantly reversed in the PC + NaHS group (p<0.05). Moreover, the beneficial effects of PC + HS was significantly (p<0.05) greater that those of NaHS alone. And again the beneficial effects of PC + NaHS were blocked by AG1478, PD98059, or LY294002.

### H_2_S upregulates ERK1/2 activity via HB-EGF/EGFR signaling in aged H9C2 cells

**Figure 5:**
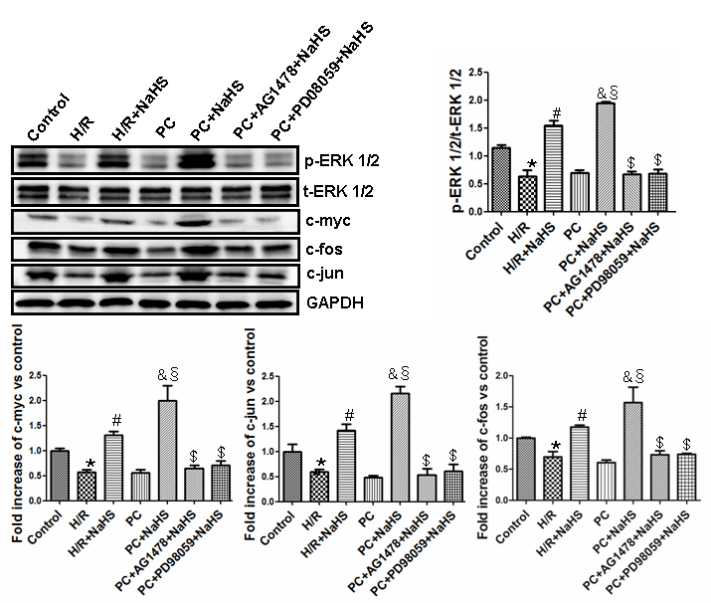
**Exogenous H_2_S up-regulates the ERK1/2 pathway by activating the HB-EGF/EGFR pathway in the aged H9C2 cells.** The graphs represent the optical density of the bands of phosphorylated ERK1/2 (p-ERK1/2) normalized to the expression of total ERK1/2 (t-ERK1/2). The graphs represent the optical density of the bands of c-myc, c-fos and c-jun normalized to the expression of the GAPDH signal. The expression levels in the control group were considered the basal levels, and the others are expressed as fold change from the control group. All data are the means ± S.E.M. of three determinations. * p<0.05 *vs*. control group; # p<0.05 *vs*. H/R group; & p<0.05 *vs*. PC group; § p<0.05 *vs*. H/R + NaHS group; $ p<0.05 *vs*. PC + NaHS group.

The activity of phosphorylated (phospho)-ERK1/2 and expression of the proto-oncogene transcription factors c-myc, c-fos and c-jun were significantly (p<0.05) lower in aged H9C2 cardiomyocytes exposed to H/R than untreated control cells, and similar suppression was seen the PC group (Figure 5). Addition of NaHS to the H/R-exposed cells significantly (p<0.05) increased phospho-ERK1/2 activity and proto-oncogene expression in both the H/R + NaHS and PC + NaHS groups, but the increases were significantly (p<0.05) greater in the PC + NaHS group. The effects of NaHS were blocked by the ERK1/2 inhibitor PD98059 or the EGFR antagonist AG1478. Although its activity varied, the total amount of ERK1/2 did not differ among the different treatment conditions (Figure 5).

### H_2_S activates the PI3K-Akt-GSK3β pathway by enhancing HB-EGF/EGFR signaling in aged H9C2 cells

**Figure 6:**
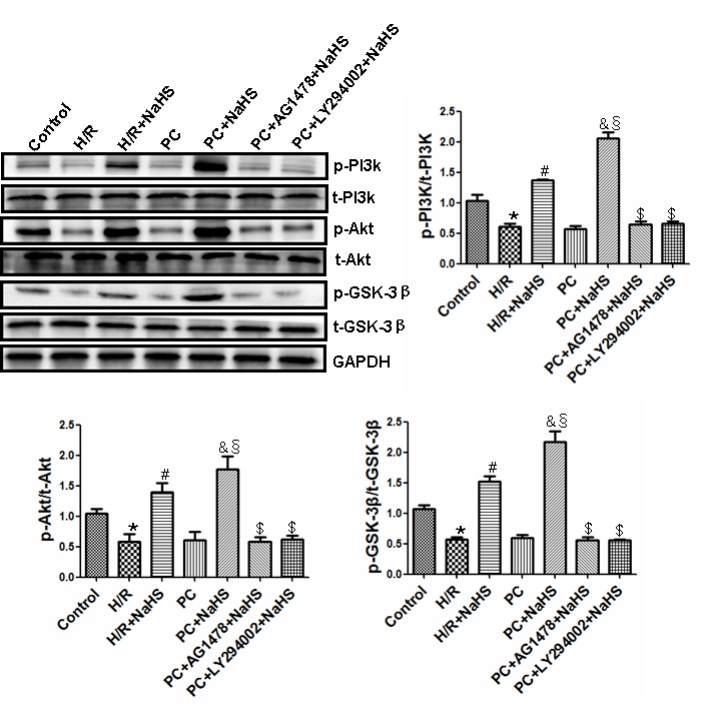
**Exogenous H_2_S activities of the PI3K-Akt-GSK-3β pathway up-regulates the HB-EGF/EGFR pathway in the aged H9C2 cells.** The phosphorylation of PI3K, Akt and GSK-3β was detected using western blotting analysis. The graphs represent the optical density of the bands of phospho-PI3K (p- PI3K), Akt (p-Akt) and GSK-3β (p- GSK-3β) normalized to the expression of total-PI3K (t-PI3K), Akt (t-Akt) and GSK-3β (t-GSK-3β). All data were from three independent experiments. * p<0.05 *vs*. control group; # p<0.05 *vs*. H/R group; & p<0.05 *vs*. PC group; § p<0.05 *vs*. H/R + NaHS group; $ p<0.05 *vs*. PC + NaHS group.

Levels of phosphorylated PI3K, Akt and GSK-3β were lower (p<0.05) in the H/R group than in the control group (Figure 6). Compared with the H/R group, levels of phosphorylated PI3K, Akt and GSK-3β were significantly (p<0.05) increased in cells treated with H/R + NaHS. The reductions in phosphorylated PI3K, Akt and GSK-3β seen in the PC group were similar to those in the H/R-only group, but the levels were significantly increased by addition of NaHS. In addition, levels of phosphorylated PI3K, Akt and GSK-3β were significantly (p<0.05) higher in the PC + NaHS group than the H/R + NaHS. These beneficial effects of NaHS were blocked by the PI3K inhibitor LY294002 or AG1478. The total amounts of PI3K, Akt and GSK-3β detected did not differ among the treatments (Figure 6).

### EGFR knockdown cancels the effect of beneficial effects of exogenous H_2_S in the aged H9C2 cells

**Figure 7:**
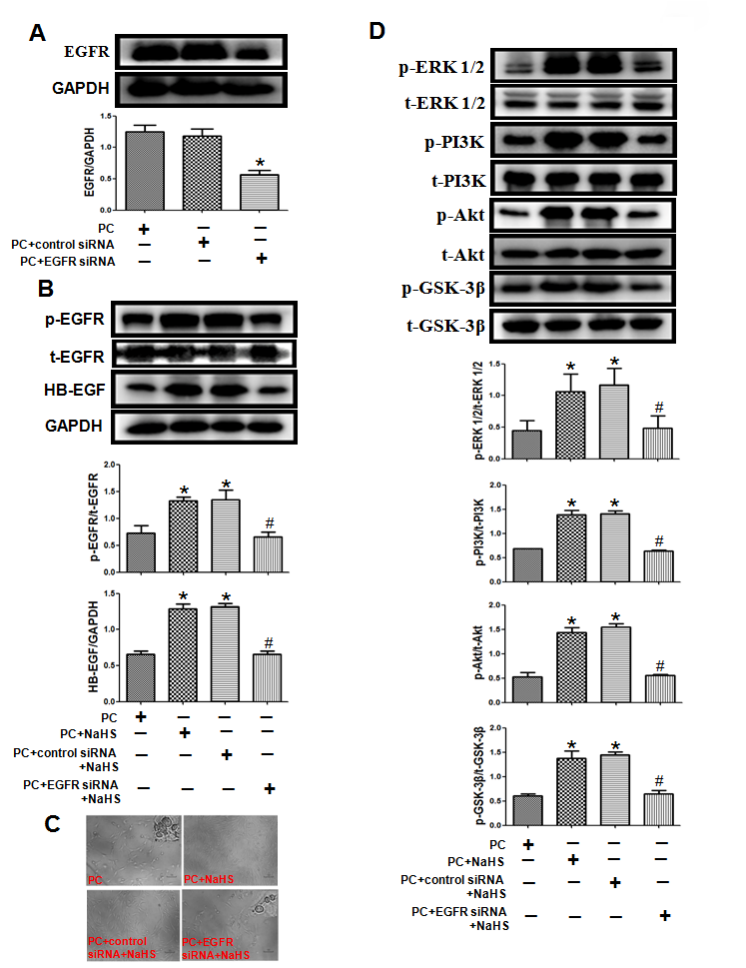
**Knockdown of EGFR cancels the effect of exogenous H_2_S on cell damage and related signaling pathways in the aged H9C2 cells.** (**A**) The knockdown of EGFR by EGFR-specific siRNA (EGFR siRNA) in the aged H9C2 cells. The cells were transfected with 50 nM EGFR siRNA or negative control siRNA (control siRNA) for 48 h during P(C) The data are the means ± S.E.M. of 3 determinations. * p<0.05 *vs*. PC + control siRNA group. (**B**) The knockdown of EGFR inhibited NaHS-increased expression of the HB-EGF and the activity of phosphorylated EGFR. The graphs represent the optical density of the bands of phosphorylated EGFR (p- EGFR) normalized to the expression of total EGFR (t-EGFR). The graphs represent the optical density of the bands of HB-EGF normalized to the expression of GAPDH signal. All data were from three independent experiments. * p<0.05 *vs*. PC group; # p<0.05 *vs*. PC + control siRNA + NaHS group. (**C**) The knockdown of EGFR cancelled NaHS-decreased cell damage. The cells were cultured in glass-bottom dishes and observed using a general inverted microscope (magnification ×100). (**D**) The knockdown of EGFR inhibited NaHS-up-regulated the ERK1/2 and PI3K-Akt-GSK-3β pathways. The phosphorylation of ERK1/2, PI3K, Akt and GSK-3β was detected using western blotting analysis. The graphs represent the optical density of the bands of phospho-ERK1/2 (p-ERK1/2), PI3K (p- PI3K), Akt (p-Akt) and GSK-3β (p-GSK-3β) normalized to the expression of total-ERK1/2 (t-ERK1/2) PI3K (t-PI3K), Akt (t-Akt) and GSK-3β (t-GSK-3β). All data were from three independent experiments. * p<0.05 *vs*. PC group; # p<0.05 *vs*. PC + control siRNA + NaHS group.

To further confirm whether that H_2_S restores PC-induced cardioprotection by up-regulating HB-EGF/EGFR signaling, EGFR was knocked down using targeted siRNA in aged H9C2 cells (Figure 7A). Knocking down EGFR inhibited the beneficial effects of NaHS on cell damage and the HB-EGF/EGFR, ERK1/2 and PI3K-Akt-GSK3β signaling summarized above (Figure 7B-D). The total amounts of EGFR, ERK1/2, PI3K, Akt and GSK-3β all remained unchanged under the different treatment conditions.

## DISCUSSION

We previously analyzed senescence-associated β-gal (SA β-gal) activity, advanced glycation end products (AGEs) content, expression of cell cycle proteins (cyclin D1 and p21^Cip/WAF−1^), and caspase-3 activity in H9C2 cells treated for 2 h with different concentrations of H_2_O_2_ and then cultured for 3 days [25]. We found that 30 μM H_2_O_2_ induces senescence but not apoptosis. We therefore used that protocol to produce an aged H9C2 cardiomyocyte model.

It is well established that PC can decrease I/R injury [22–29]. With aging, however, PC reportedly loses its ability to protect cells against apoptosis and oxidative stress due to changes in mRNA and protein expression profiles [5–7]. Our earlier studies found that the loss of protection by PC in aged cardiomyocytes is associated with decreased endogenous production H_2_S but that exogenous H_2_S restored the protective effects of PC by inhibiting oxidative stress, endoplasmic reticulum stress and mPTP opening while upregulating autophagy [22–27]. The underlying mechanisms by which H_2_S restored the cardioprotective effects of PC are not fully understood, however.

HB-EGF is highly expressed in the lung, heart, brain and skeletal muscle, and is involved in such physiological and pathological processes as blastocyst implantation, wound healing, atherosclerosis, tumor growth and smooth muscle hyperplasia [30,31]. EGFR is an important transmembrane receptor [31], and the HB-EGF/EGFR complex plays a key role in cell cycling, migration, proliferation, differentiation, and apoptosis [11,31]. In addition, upregulation of HB-EGF/EGFR signaling prevents cardiac I/R injury by improving cell function, decreasing infarct size, and inhibiting apoptosis, among other mechanisms [32]. In the present study, we found that H/R decreased levels of HB-EGF and phospho-EGFR in aged H9C2 cardiomyocytes with or without PC. However, treatment with NaHS significantly increased levels of both HB-EGF and phospho-EGFR and restored the protective effects of PC. These effects were blocked by the EGFR inhibitor AG1478, indicating that exogenous H_2_S restored PC-mediated cellular protection in aged cardiomyocytes by upregulating HB-EGF/EGFR signaling.

It is known that H/R (or I/R) causes cell damage resulting in leakage of LDH and CK from cells. Cyt-c is released from damaged mitochondria during H/R (or I/R), which initiates the mitochondrial apoptosis pathway [26]. Once released, Cyt-c triggers cytosolic caspase-3 activation through formation of the cytochrome c/Apaf-1/caspase-9 complex, or apoptosome, which leads to apoptosis [23–26]. Bcl-2 is a potent inhibitor of apoptosis that inhibits mitochondrial disruption, the subsequent Cyt-c release, and caspase activation [23–26]. PC prevents myocardial I/R injury by decreasing oxidative stress, leakage of myocardial enzymes (LDH, CK, etc.), the incidence of apoptosis, and infarct size, thereby improving cardiac function [22–27]. But these beneficial effects are diminished in the aged heart. We demonstrated here that H/R increases cell damage, LDH and CK activities, the incidence of apoptosis, and the expression of apoptosis-related factors (Cyt-c, caspase-3,-9, Bcl-2) in aged cardiomyocytes. Although PC did not protect against H/R injury in aged cardiomyocytes, addition of NaHS led to significant reductions in cell damage, LDH and CK activities, expression of pro-apoptotic related factors (Cyt-c, caspase-3,-9), and the incidence of apoptosis. It also increased expression of the anti-apoptotic factor Bcl-2. These effects were blocked by AG1478, further confirming that exogenous H_2_S contributes to the PC-mediated protection of aged cardiomyocytes by activating HB-EGF/EGFR signaling.

The HB-EGF/EGFR complex activates multiple signaling pathways in cells, including the ERK1/2 and PI3K-Akt-GSK-3β pathways, which play key roles in cell proliferation, differentiation, chemotaxis, and apoptosis [15]. ERK1/2 in turn mediates activation of the proto-oncogene transcription factors c-myc, c-fos and c-jun [33]. HB-EGF/EGFR also reportedly promotes atherosclerosis by increasing vascular smooth muscle cell proliferation via upregulation of ERK1/2 and PI3K-Akt signaling [34] and contributes to myocardial hypertrophy by promoting fibroblast proliferation via the ERK1/2 pathway [12]. El-Assal et al. reported that HB-EGF/EGFR inhibits myocardial I/R injury through phosphorylation of ERK1/2 and PI3K [32]. Our results showed that H/R downregulated ERK1/2-c-myc (and c-fos and c-jun) and PI3K-Akt-GSK-3β signaling, and that PC did not affect these pathways in aged cardiomyocytes. By contrast, treatment with NaHS led to increased activity in the ERK1/2-c-myc (and c-fos and c-jun) and PI3K-Akt-GSK-3β pathways in cells, with and without PC. The beneficial effects of NaHS were suppressed by knocking down EFGR using targeted siRNA or by treating cells with the EFGR antagonist AG1478, the ERK1/2 inhibitor LY294002, or the PI3K inhibitor PD98059. This suggests exogenous H_2_S restores PC-mediated protection against I/R in aged cardiomyocytes by activating the HB-EGF/EGFR pathway via phosphorylation of its downstream mediators, including components in the ERK1/2-c-myc (and c-fos and c-jun) and PI3K-Akt-GSK-3β pathways.

**Figure 8:**
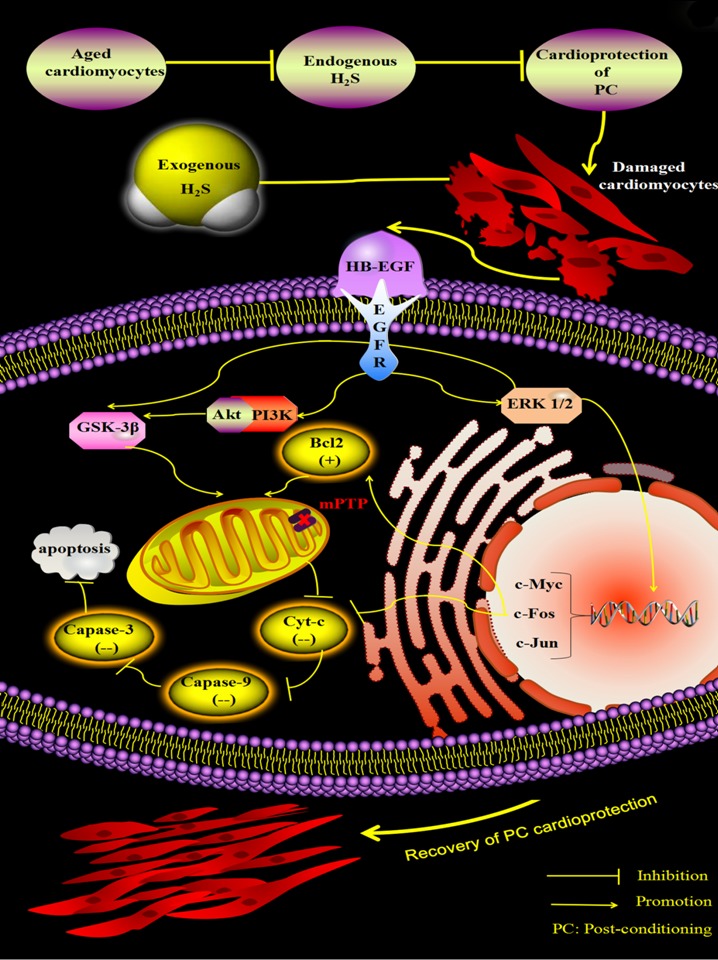
**Exogenous H_2_S restores PC-induced cardioprotection by up-regulating the HB-EGF/EGFR pathway, which activates the ERK1/2-c-myc (and fos and c-jun) and PI3K-Akt-GSK-3β pathways in the aged cardiomyocytes.** Endogenous H_2_S is decreased and then leads to loss of the protective role of PC in the aged cardiomyocytes. Supplementation of exogenous H_2_S up-regulates the HB-EGF/EGFR pathway, which activates the ERK1/2-c-myc (and fos and c-jun) and PI3K-Akt-GSK-3β pathways, which inhibits apoptosis via down-regulating the mitochondrial apoptosis pathway (Cyt c-caspase-3) by decreasing the release of Cyt-c and the expression of caspase-3, and -9 and increasing the expression of Bcl-2. Finally, PC-induced cardioprotection is restored.

In summary, our results suggest that exogenous H_2_S restores PC-mediated protection of aged cardiomyocytes by upregulating HB-EGF/EGFR signaling, which in turn upregulates activity in the ERK1/2-c-myc (and fos and c-jun) and PI3K-Akt-GSK-3β pathways in the aged cardiomyocytes (Figure 8). These findings provide insight into the mechanisms underlying recovery of the beneficial effects of PC in aged hearts treated with exogenous H_2_S and opens a window for innovative H_2_S-based treatment strategies for aged patients with ischemic cardiovascular diseases.

## MATERIALS AND METHODS

### Materials and drugs

Sodium hydrogen sulfide (NaHS), H_2_O_2_, PD98059 (an inhibitor of ERK1/2), LY294002 (an inhibitor of PI3K) and AG1478 (an inhibitor of EGFR) were purchased from Sigma Chemical Co. (St. Louis, MO, USA). The primary antibodies for anti-cleaved caspase-3 and -9, Bcl-2, cytochrome *c* (Cyt-*c*), c-myc, c-jun, c-fos and GAPDH were from Proteintech (Wuhan, China). The anti-ERK1/2, p-ERK1/2, PI3K, p-PI3K, GSK-3β, p-GSK-3β, Akt, p-Akt, HB-EGF, EGFR and p-EGFR antibodies were obtained from Cell Signaling Technology (Denver, CO, USA). Assay kits for lactate dehydrogenase (LDH) and creatine kinase (CK) were purchased from Nanjing Jiancheng Bioengineering Institute (Nanjing, China). The Cell Counting Kit-8 (CCK-8) was obtained from Boster Bio-engineering Limited Company (Wuhan, China). The HB-EGF ELISA kit was purchased from Elabscience Biotechnology (Wuhan, China). All other chemicals were from Sigma or Santa Cruz.

### Culture of H9C2 cells

The rat cardiomyocyte line H9C2 cells were purchased from the American Type Culture Collection and were cultured in growth medium DMEM containing 10% foetal bovine serum (FBS), 100 U/ml penicillin, and 100 mg/ml streptomycin. The experiments were performed when the cells reached 70-80% confluence by trypsinization according to the standard procedures and the medium was changed every 48 h.

### The aged H9C2 cells induced by H_2_O_2_ and the established H/R model

The treatment for H_2_O_2_ induction was as previously described [25]. Briefly, the DMEM supplemented with 10% FBS was removed, and DMEM supplemented with 30 μM H_2_O_2_ was added to the H9C2 cells in the culture cluster for 2 h and subsequently cultured for 72 h.

A hypoxic condition was produced by D-Hank’s solution saturated with 95% N_2_ and 5% CO_2_. The pH was regulated to 6.8 with lactate to mimic the ischemic solution. The aged H9C2 cells were put into a hypoxic incubator that was equilibrated with 1% O_2_/5% CO_2_/94% N_2_. After hypoxia, the culture medium was rapidly replaced with fresh DMEM with 10% foetal bovine serum (normoxic culture solution) for initiating reoxygenation.

### Experimental groups

The aged H9C2 cells were randomly divided into the following 8 groups and each group included 8 samples (n = 8): (1) Control group (control): The aged H9C2 cells were cultured for 9 h with 10% FBS-DMEM. (2) H/R group: The aged H9C2 cells were exposed to hypoxic culture medium for 3 h and reoxygenated for 6 h by replacing the hypoxic culture medium with fresh DMEM with 10% foetal bovine serum. (3) H/R + NaHS group: The procedure was similar to that for group 2, except that 100 μM NaHS was added in 6 h of reoxygenation. (4) PC group: At the end of 3 h of hypoxia, the aged H9C2 cells were exposed to normoxic culture solution for 5 min, after which the cells were placed in hypoxic solution for 5 min. The PC cycle was repeated 3 times and followed by 6 h of reoxygenation. (5) PC + NaHS group: At the end of 3 h of hypoxia, initiated immediately at the onset of reoxygenation, 100 μM NaHS was given at the onset of reoxygenation for 5 min followed by 5 min of hypoxia. This protocol was repeated twice. The cells were then treated similar to those of group 3. (6) PC + AG1478 (or PD98059, or LY294002) + NaHS group: 100 nM AG1478 (or 10 µM PD98059 or 10 µM LY294002) was added to the medium 40 min before the end of hypoxia. The cells were then treated similar to those of group 5.

### Cell viability assay

Cells were seeded in 96-well plates. After 6 h of each treatment, the cell counting kit-8 (CCK-8) was added to each well according to the manufacturer's introductions. Optical density was measured by a microplate spectrophotometer at a wavelength of 570 nm (A570).

### Live cells imaging

The cells were cultured in glass-bottomed dishes and observed using the general inverted microscope.

### Measurement of LDH and CK activities

Measurements of LDH and CK activities were made as previously described [4,24]. Assay kits for LDH and CK were purchased from Nanjing Jiancheng Bioengineering Institute (Nanjing, China). The activities of LDH and CK from all experimental groups in the aged H9C2 cells culture medium were determined by a commercially available assay kit according to the manufacturer's instructions. The enzyme product was measured spectrophotometrically at a wavelength of 340 nM.

### Detection of HB-EGF content by ELISA

The concentration of HB-EGF in the cell culture medium was determined by an HB-EGF ELISA kit as described previously [35]. First, 100 μl of each standard or sample was added to the wells at 37°C for a 1 h incubation. Then the liquid was removed and replaced with 100 μl of Biotinylated Detection Ab at 37°C for a 1 h incubation. The liquid was aspirated and washed 3 times. After this, 100 μl of HRP conjugate was added and incubated at 37°C for 30 minutes. The liquid was aspirated and washed 5 times. Then, 90 μl of substrate reagent was added at 37°C for a 15 min incubation, and finally 5 μl of stop solution was added. The OD was read at 450 nm immediately.

### Analysis of apoptosis by flow cytometry

The apoptotic rate was measured by flow cytometry with annexinV-FiTC/PI double staining as described previously [36]. The apoptotic rate was detected by flow cytometry using the FITC Annexin V apoptosis detection kit I (BD Biosciences, San Jose, CA, USA). Cells were washed with cold PBS and re-suspended in binding buffer at a concentration of 1 × 10^6^ cells/ml. A total of 100 μl of the solution was transferred to a 5 ml culture tube and 5 μl of FITC-Annexin V and 5 μl of PI were added. The cells were incubated for 15 min at room temperature in the dark, and 400 μl of binding buffer was added to each tube. The fluorescence was analysed by flow cytometry. The percentage of apoptotic cells was determined using the Mod Fit LT software (Verity Software House Inc., Topsham, ME, USA).

### siRNA transfection

Transfection of H9C2 cells by EGFR siRNA and corresponding control siRNA was achieved by using the Lipofectamine^TM^ 3000 transfection agent from Invitrogen (Burlington, ON) as described previously [21]. In brief, H9C2 cells were seeded at an equal number of cells (2.0×10^5^ per plate) in 60 mm^2^ plates in the medium containing 10% FBS. The cells were plated to form 60-70% confluent monolayers for siRNA transfection. siRNA and the transfection reagent complex were added to the serum-free medium for 4 h, and the transfection continued for another 48 h in serum-containing regular medium. After that, the cells were collected for detection of protein expressions with western blotting analysis.

### Western blot analysis

Protein expression was measured by western blotting as described previously [27]. Briefly, equal amounts of proteins were subjected to sodium dodecyl sulphate polyacrylamide gel electrophoresis and blotted on polyvinylidene fluoride membranes. The membranes were incubated with the primary antibodies. The secondary antibody was goat anti-rat immunoglobulin G. The intensities of the protein bands were quantified by a Bio-Rad ChemiDoc™ EQ densitometer and Bio-Rad Quantity One software (Bio-Rad Laboratories). The protein concentration was quantified using the BCA Protein Assay kit (Beyotime, Nantong, China).

### Detection of Cyt-c release from mitochondrial

Western blot analysis of Cyt-c in the cytosolic fraction was performed as described previously [27]. Briefly, cells were harvested, washed twice with ice-cold PBS, and incubated in ice-cold Tris-sucrose buffer (0.35 mM sucrose, 10 mM Tris-HCl at pH 7.5, 1 mM EDTA, 0.5 mM dithiothreitol, 0.1 mM phenylmethylsulphonyl fluoride). After a 40 min incubation, cells were centrifuged at 1000 × g for 5 min at 4°C and the supernatant was then centrifuged at 40,000 × g for 30 min at 4°C. The supernatant was retained as the cytosolic fraction and analysed by western blotting with a primary rat anti-Cyt-c monoclonal antibody and a secondary goat anti-rat immunoglobulin G. GAPDH expression was used as the control.

### Statistical analysis

All data are expressed as the mean±SE and represent at least three independent experiments. Statistical comparisons were made using Student’s *t*-test or one-way ANOVA followed by a post hoc analysis (Tukey test) where applicable. The significance level was set at p<0.05.
